# ﻿A rare chromosomal polymorphism in a Kangayam bull (*Bosindicus*) of south India

**DOI:** 10.3897/CompCytogen.v15.i4.71295

**Published:** 2021-12-15

**Authors:** Vemula Harshini, P. Kumarasamy, S.M.K. Karthickeyan

**Affiliations:** 1 Department of Animal Genetics and Breeding, Madras Veterinary College, Tamil Nadu Veterinary and Animal Sciences University, Chennai-600 007, Tamil Nadu, India Tamil Nadu Veterinary and Animal Sciences University Chennai India

**Keywords:** Heterochromatin variation, individual chromosomal polymorphism, karyological screening

## Abstract

A chromosomal polymorphism was detected on karyological screening of Kangayam breeding sires prior to subjecting them for frozen semen collection. One bull possessed the chromosomal complement 2n = 60, consisting of 58 acrocentric autosomes, one large sub-metacentric X-chromosome, and one small acrocentric Y-chromosome with a small visible p-arm, which was further confirmed using CBG- and GTG-banding. This polymorphism was attributed to a heterochromatin variation of the acrocentric Y-chromosome routine in the *Bosindicus* Linnaeus, 1758 cattle.

## ﻿Introduction

The Kangayam breed of cattle is a pride of Tamil Nadu and native to south India. It is well known for its excellent draught qualities, adaptation to poor nutrition and longevity (Kandasamy 2001). Despite large scale transformation and decline in agricultural practices, there is still a demand for the Kangayam cattle from the neighboring states such as Kerala, Karnataka and Andhra Pradesh. The breed has been transported to countries like Brazil, Malaysia, Philippines and Sri Lanka. Given its importance, the breed has been characterized phenotypically as well as through cytogenetic (Kumarasamy et al. 2006) and microsatellite analyses (Karthickeyan et al. 2009).

In the process of evolution, chromosomes have undergone rearrangements and form species-specific karyotypes. Iannuzzi and Di Meo (1995) stated that centric fusion translocations, and peri- or paracentric inversions along with the loss or gain of heterochromatin appeared to be the main chromosomal rearrangements occurred and thus differentiated the chromosomal complements across the bovid species. The variations in the size of the Y-chromosomes among subspecies are proportional to the amount of heterochromatin present (Cabelova et al. 2012). It shows the involvement of heterochromatin in the karyotype evolution of taxa of higher as well as lower ranks.

The chromosomal complements of *Bosindicus* Linnaeus, 1758 and *Bostaurus* Linnaeus, 1758 cattle are highly similar except for the Y-chromosome morphology being acrocentric (one arm) and sub-meta/metacentric (two arms), respectively. This morphological difference is due to the pericentric inversion which might have been occurred at the time of divergence (Goldammer et al. 1997; Di Meo et al. 2005). In the present study, a phenotypically healthy Kangayam bull was found carrying a different type of Y-chromosome on routine karyological screening, which was subjected to banding for unequivocal identification of chromosome morphology.

## ﻿Material and methods

A total of 46 blood samples of Kangayam bulls were received for routine cytogenetic screening before using them for semen collection (14 from Buffalo Frozen Semen Station, 16 from Kangayam Cattle Research Station and 16 from field progeny tested animals in Tamil Nadu).

Metaphase chromosomes were obtained using short term lymphocyte culture technique (Moorhead et al. 1960), standardized with minor modifications in the Cytogenetics Laboratory of Department of Animal Genetics and Breeding, Madras Veterinary College, Chennai, India. The chromosome spreads were examined under Olympus microscope (BX61, USA) and more than 200 metaphases were screened. The good metaphase spreads were photographed using applied spectral imaging software. The cell fixation from one Kangayam bull carrying an unusual Y-chromosome was subsequently further studied using different cytogenetic banding techniques.

The chromosome preparations were CBG-banded using barium hydroxide [Ba(OH)_2_] treatment as described by Sumner et al. (1972) with minor modifications (Harshini et al. 2020). GTG-banding technique was done as described by Seabright (1971) with modifications in concentration of the trypsin and exposure time. Slides aged for 5–7 days were immersed in Sorenson’s phosphate buffer for 2–3 seconds and transferred to 0.025 per cent trypsin solution for 10–14 seconds. Then the slides were immediately transferred to Sorenson’s phosphate buffer to stop the action of trypsin, washed twice in double distilled water and subsequently air-dried. The chromosomes were stained with 4 per cent Giemsa for 15 minutes and rinsed thoroughly in two consecutive washes in double distilled water. The chromosomes were then observed under microscope, photographed and karyotyped.

## ﻿Results and discussion

**Figure 1.**
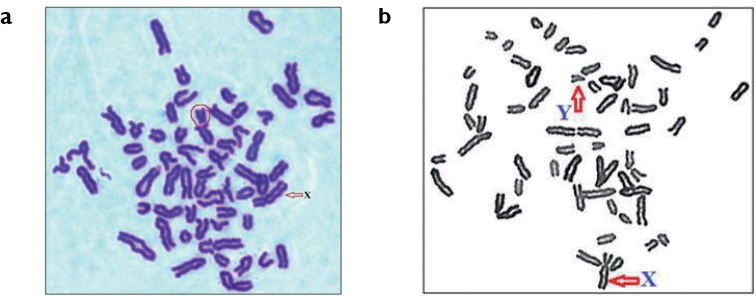
Giemsa-stained metaphase spreads of Kangayam bulls showing general similarity of acrocentric autosome set and X-chromosome, and different morphology of Y-chromosome: subacrocentric with small p-arm (**a**, encircled) and typical acrocentric (**b**).

All the Kangayam bulls, except the one investigated in detail, found to have 60 chromosomal diploid set with 29 pairs of acrocentric autosomes, one large sub-metacentric X- and the smallest acrocentric Y-chromosome. One bull showed similar chromosomal profile except for the presence of an unpaired smallest subacrocentric chromosome possessing a small p-arm (Fig. 1) in all the spreads screened. A similar kind of chromosomal complement was reported earlier by Iannuzzi et al. (2001) in Chainina cattle (*Bostaurus*) and considered as a sex-autosomal reciprocal translocation between the chromosomes Y and 9. Therefore, this Kangayam sample was further investigated using CBG- and GTG-banding techniques.

**Figure 2.**
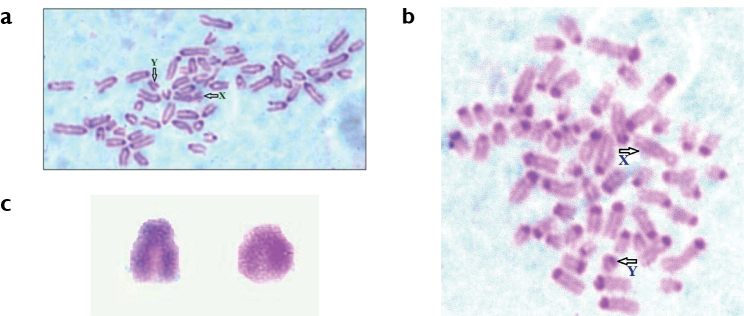
CBG-banded metaphase spreads of Kangayam bulls (**a, b**) bearing C-positive heterochromatic Y chromosome of two alternative types - subacrocentric (**c**, left) or acrocentric (**c**, right).

CBG-banding revealed that all the acrocentric chromosomes each showed characteristically a positive C-band as a distinctly-stained centromeric region. The X-chromosome was stained lightly across its entire length (Fig. 2). This is like the standard CBG-banding pattern of cattle as reported for Red Danish (Hansen 1973), *Bostaurus* L. (Iannuzzi and Berardino 1985), Jersey crossbreds (Chauhan et al. 2009), Tho-Tho cattle (Longkumer et al. 2015), mithun (Ezung 2016) and Nellore cattle (Amancio et al. 2019). However, the unpaired acrocentric chromosome with a small p-arm also stained darkly throughout the length showing no centromere banding (Fig. 2). Thus, it was confirmed as a sex chromosome (Y) and its small extra p-arm was not a translocated portion of any autosome which arms are lightly C-stained.

**Figure 3.**
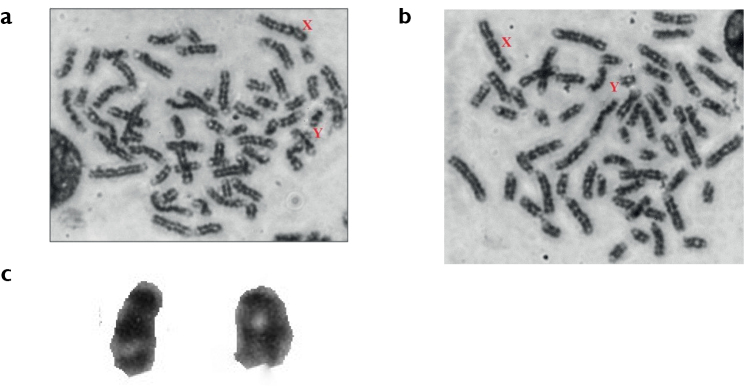
GTGbanded metaphase spreads of Kangayam bulls (**a**, **b**) with different Y-chromosome showing visible dark band in p-arm (**c,** left) and no prominent p-arm (**c**, right).

Upon GTG-banding, the Y-chromosome displayed a rearrangement in the distribution of G-bands divided for two arms (p, q) in the subacrocentric Y-chromosome and being situated together in the one arm (q) of acrocentric structure (Fig. 3), which is comparable to the standard G-banding pattern of *Bosindicus* cattle *viz.* Red Kandhari (Katkade 2005), Khillari (Nakod 2013), Malnad Gidda (Suresh et al. 2015), Indonesian native bulls (Ciptadi et al. 2017) and Nellore cattle (Amancio et al. 2019). The GTG-banding results also confirmed that there was no translocation between autosomes and Y-chromosome, as all the autosomes were having the typical G-banding pattern, as those of Iannuzzi and Di Meo (1995) and ISCNDB 2000 (Cribiu et al. 2001).

Of many studies pertaining to variations in morphology of cattle Y-chromosome, only a few cytogeneticists detected the polymorphisms. Halnan and Watson (1982) studied the Y-chromosome of *Bosindicus* breeds Sahiwal, Sindhi, Brahman, Santa Gertrudis and Belmont Red, derived from Zebu males, and reported as acrocentric though the centromere was found at a variable distance from the terminal point of the small p-arm; sometimes there would be no visible chromatin beyond the centromere and at other times the Y-chromosome would have distinct p-arms. Further, they also found visible subtelocentric Y-chromosome in every Sahiwal bull studied which they considered as differing only insignificantly from acrocentric according to the definition of Potter and Upton (1979) or Potter et al. (1979). According to them, these points served to raise the question of possible Y-chromosome polymorphism in *Bosindicus* in contradiction to the relative stability of the Y-chromosome in *Bostaurus*. Iannuzzi and Di Meo (1995) noticed that even though, there are size and morphological differences in the sex chromosomes (X and Y), R-banding patterns are conserved among cattle, river buffalo, sheep and goat. The differences are due to peri/paracentric inversions in sex chromosomes with loss or gain of heterochromatin.

In the present case also, there is a distinct p-arm in Y-chromosome of a Kangayam bull, when other bulls were possessing acrocentric Y-chromosomes with no prominent p-arm. As for the criteria for chromosome classification, the terms ‘acrocentric’ and ‘subtelocentric’ belong to different nomenclature systems, and their common use in the description of the same karyotype should be incorrect. Subtelocentric means the presence of telocentric, that is the chromosomes being strictly one-armed. It has been known from classic cytogenetics that the centromere is always distanced, at least minimally (very short arm) from the side opposite to the main (long) arm. Because of some uncertainty in definition of arm proportions in small chromosomes, such as the Y-chromosome of many mammals, including bovid taxa, and to stress a disproportion in arms of Y-chromosomes of the bulls studied, the term “subacrocentric” for the Y variant with a visible p-arm should be appropriate in recognition to the typical acrocentric of other breeding sires.

This visible p-arm appears attributable to heterochromatin variation and this can be considered as a possible Y-chromosomal polymorphism in Kangayam bull of south India. Even though, it is not a chromosomal abnormality, the productive and reproductive performance of the bull and its descendents should be studied to know the effect of the subacrocentric Y-chromosome; if the bull is allowed to breed without being usually culled upon receipt of the screening results.

## References

[B1] Amancio AP Duarte SSM Silva RC da Cruz AS Silva DC da Silva CC da Cruz AD (2019) Banded karyotype of Nellore cattle (*Bostaurusindicus Linnaeus*, 1758). Comparative Cytogenetics 13(3): 265–275. 10.3897/CompCytogen.v13i3.36449 31558984 PMC6728309

[B2] Cabelova K Kubickova S Cernohorska H Rubes J (2012) Male speciﬁc repeats in wild Bovidae. Journal of Applied Genetics 53: 423–433. 10.1007/s13353-012-0108-y 22895838

[B3] Chauhan JB Patel RK Singh KM Soni KJ (2009) A dicentric Robertsonian translocation, rob (1; 29) in Indian Jersey crossbred (*Bostaurus* × *Bosindicus*) bull. Nucleus 52: 119–123.

[B4] Ciptadi G Ihsan MN Nurgiartiningsih VMA Ardyah IP Mudawamah M (2017) The normal karyotyping result of Indonesian native breed bull qualified for artificial insemination. Biodiversitas 18(4): 1462–1467. 10.13057/biodiv/d180423

[B5] Cribiu EP Di Berardino D Di Meo GP Eggen A Gallagher DS Gustavsson I Hayes H Iannuzzi L Popescu CP Rubes J Schmutz S Stranzinger G Vaiman A Womack J (2001) International system for chromosome nomenclature of domestic bovids (ISCNDB 2000). Cytogenetics and Cell Genetics 92(34): 283–299. 10.1159/000056917 11435702

[B6] Di Meo GP Perucatti A Floriot S Incarnato D Rullo R Caputi Jambrenghi A Ferretti L Vonghia G Cribiu E Eggen A Iannuzzi L (2005) Chromosome evolution and improved cytogenetic maps of the Y chromosome in cattle, Zebu, River buffalo, sheep and goat. Chromosome Research 13(4): 349–355. 10.1007/s10577-005-2688-4 15973500

[B7] Ezung NM (2016) Comparative cytogenetic studies of Mithun and Mithun X Cattle Cross. M.V.Sc. thesis, Maharashtra Animal and Fishery Sciences University, Nagpur, 102 pp.

[B8] Goldammer T Brunner RM Schwerin M (1997) Comparative analysis of Y-chromosome structure in *Bostaurus* and *Bosindicus* by FISH using region specific, microdissected, and locus specific DNA probes. Cytogenetic and Genome Research 77(3–4): 238–241. 10.1159/000134584 9284924

[B9] Halnan CRE Watson JI (1982) Y chromosome variants in cattle *Bostaurus* and *Bosindicus*. Annales de Génétique et de Sélection Animale 14(1): 1–16. 10.1186/1297-9686-14-1-1 22896221 PMC2733894

[B10] Hansen KM (1973) Heterochromatin (C-bands) in bovine chromosomes. Hereditas 73(1): 65–69. 10.1111/j.1601-5223.1973.tb01068.x 4142027

[B11] Harshini V Kumarasamy P Karthickeyan SMK Cauveri D Gowri AM Rangasamy S (2020) Ascertaining the paternal lineage in crossbred calves. Journal of Genetics 99(1): 1–3. 10.1007/s12041-020-01193-y 32482916

[B12] Iannuzzi L Di Berardino D (1985) Diagrammatic representation of RBA-banded chromosomes of swamp buffalo (*Bubalusbubalis* L.) and sex chromosome banding homologies with cattle (*Bostaurus* L.). Caryologia 38(3–4): 281–295. 10.1080/00087114.1985.10797751

[B13] Iannuzzi L Di Meo GP (1995) Chromosomal evolution in bovids: a comparison of cattle, sheep and goat G-and R-banded chromosomes and cytogenetic divergences among cattle, goat and river buffalo sex chromosomes. Chromosome Research 3(5): 291–299. 10.1007/BF00713067 7551543

[B14] Iannuzzi L Molteni L Di Meo GP De Giovanni A Perucatti A Succi G Incarnato D Eggen A Cribiu EP (2001) A case of azoospermia in a bull carrying a Y-autosome reciprocal translocation. Cytogenetics and Cell Genetics 95(3–4): 225–227. 10.1159/000059349 12063403

[B15] Kandasamy N (2001) Kangayam breed of cattle: present status and management practices. Proceedings of the Workshop on Indigenous Cattle and Their Role in the New Millennium. Erode, March, 2001. Tamil Nadu, 24–25.

[B16] Karthickeyan SMK Sivaselvam SN Selvam R Thangaraju P (2009) Microsatellite analysis of Kangayam cattle (*Bosindicus*) of Tamilnadu. Indian Journal of Science and Technology 2(10): 38–40. 10.17485/ijst/2009/v2i10.11

[B17] Katkade BS (2005) Cytogenetic Studies on Indian Domestic Cattle (*Bosindicus*). M.V.Sc. thesis, Maharashtra Animal and Fishery Sciences University, Nagpur, India, 40 pp.

[B18] Kumarasamy P Sivaselvam SN Thara S Thangaraju P Nainar AM (2006) Chromosomal studies on Kangayam cattle. Indian Veterinary Journal 83(10): 1072–1073.

[B19] Longkumer I Mukherjee A Yenisetti SC Mukherjee S Mech M (2015) Complete cytogenetic insight of Tho-Tho cattle. Journal of Agriculture, Science and Techonology 5: 277–285. 10.17265/2161-6256/2015.04.006

[B20] Moorhead PS Nowell PC Mellman WJ Battips DM Hungerford DA (1960) Chromosome preparation of leucocytes cultured from human peripheral blood. Experimental Cell Research 20(3): 613–616. 10.1016/0014-4827(60)90138-5 13772379

[B21] Nakod N (2013) Karyological evaluation of Khillar cattle. M.V.Sc. thesis, Maharashtra Animal and Fishery Sciences University, Nagpur, India, 27 pp.

[B22] Potter WL Upton PC (1979) Y Chromosome morphology of cattle. Australian veterinary Journal 55: 539–541. 10.1111/j.1751-0813.1979.tb07026.x 543828

[B23] Potter WL Upton PC Blackshaw AW (1979) Presumptive 1/29 autosomal translocation in Australian cattle. Australian Veterinary Journal 55: 209–213. 10.1111/avj.1979.55.5.209 475674

[B24] Seabright M (1971) A rapid banding technique for human chromosomes. Lancet 2: 971–972. 10.1016/S0140-6736(71)90287-X 4107917

[B25] Sumner AT (1972) A simple technique for demonstrating centromeric heterochromatin. Experimental Cell Research 75: 304–306. 10.1016/0014-4827(72)90558-7 4117921

[B26] Suresh SC Nagaraja CS Satheesha GM (2015) Cytogenetic studies in Malnad Gidda cattle. Wayamba Journal of Animal Science 1424965516: 1059–1065.

